# Automated measurement and analysis of sidewall roughness using three-dimensional atomic force microscopy

**DOI:** 10.1186/s42649-022-00070-5

**Published:** 2022-03-08

**Authors:** Su-Been Yoo, Seong-Hun Yun, Ah-Jin Jo, Sang-Joon Cho, Haneol Cho, Jun-Ho Lee, Byoung-Woon Ahn

**Affiliations:** 1 Park Systems Corp., 109, Gwanggyo-ro, Yeongtong-gu, Suwon-si, 16229 South Korea; 2 Imec, Remisebosweg 1, 3001 Leuven, Belgium

**Keywords:** AFM, 3D-AFM, Measurement and analysis, Sidewall roughness, Metrology and inspection

## Abstract

As semiconductor device architecture develops, from planar field-effect transistors (FET) to FinFET and gate-all-around (GAA), there is an increased need to measure 3D structure sidewalls precisely. Here, we present a 3-Dimensional Atomic Force Microscope (3D-AFM), a powerful 3D metrology tool to measure the sidewall roughness (SWR) of vertical and undercut structures. First, we measured three different dies repeatedly to calculate reproducibility in die level. Reproducible results were derived with a relative standard deviation under 2%. Second, we measured 13 different dies, including the center and edge of the wafer, to analyze SWR distribution in wafer level and reliable results were measured. All analysis was performed using a novel algorithm, including auto flattening, sidewall detection, and SWR calculation. In addition, SWR automatic analysis software was implemented to reduce analysis time and to provide standard analysis. The results suggest that our 3D-AFM, based on the tilted Z scanner, will enable an advanced methodology for automated 3D measurement and analysis.

## Introduction

### Necessity of AFM in the semiconductor industry

Since the architectural development of integrated circuits has progressed for high-performance electrical devices, it has become necessary to measure complex 3D structures, such as FinFET structures in 3D NAND flash memories. Compared to other methodologies, atomic force microscopes can measure the topography, roughness, and structure angle through non-destructive measurement with the advantage of high-resolution measurement, reproducibility, and reliability (Barrett, 1991; Baselt & Baldeschwieler, 1993; Binning et al., 1986; Hansma et al., 1994; Meyer & Amer, 1988; Nakano, 1998). AFM is a research tool that can be applied to various fields such as materials science, life science, metrology, and the semiconductor industry. However, AFM has a weakness in measurement time, so the AFM industry has been researching how to reduce takt time.

In front-end-of-line (FEOL) EUV photolithography, the most important technology for the next generation semiconductor chip, SWR optimization and measurement become more critical as the line width scales down. Since high SWR reduces the electrical characteristics of the circuit made through the pattern, it is essential to control SWR with precise measurement methodologies (Hutchinson, 1998).

Also, in middle-end-of-line (MEOL) packaging technology, rough sidewalls (scalloped sidewall) of through silicon vias (TSV) can cause yield loss due to ineffective charging, which can lead to long-term device reliability problems. Therefore, the capabilities of 3D-AFM that can measure SWR is crucial in the semiconductor industry (Yu & Kumar, 2015).

It has been reported in International Roadmap Devices and Systems 2020 Metrology that the Scanning Electron Microscope (SEM) is a semiconductor in-line metrology device that occupies a high proportion of metrology equipment used for measuring critical dimension (CD), defects, and particle detection (International Roadmap for Devices and Systems™, Metrology, 2021). However, SEM cannot measure CD at the level of 10 nm or less due to its limitation in resolution. It also has limitations in the destructive sampling process, defects by e-beams, and the charging effect. Compared to SEM, AFM is a non-destructive metrology tool and has the advantage of measuring CD of 5 nm or less in an isolated line. In addition, AFM surface roughness measurement can accurately measure SWR.

### About 3D-AFM

One of the fundamental measurement modes in AFM is contact mode. Contact mode utilizes the AFM tip to be in contact with the sample and obtains various properties, such as resistance, elasticity, topography, conductivity, and electrostaticity. However, tip wear and sample damage may occur.

In contrast, non-contact mode obtains 3D nanostructured surface data by measuring the van der Waals forces between the tip and the sample. Non-contact mode has the advantage of preventing tip wear so that tip sharpness can be maintained throughout the measurement. Also, sample damage is minimized so that the high-resolution image of the sample can be obtained uniformly and accurately.

**Fig. 1:**
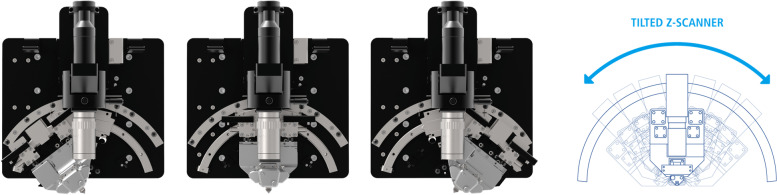
3D and 2D modeling of the Z scanner tilting motion of 3D-AFM (Park Systems Corp, 2021). The cantilever can be tilted up to 38° to either side for a total of 76°

Classic AFMs, whether using contact mode or non-contact mode, only measure samples perpendicularly and have limitations in measuring 3D information of sidewalls. Unlike normal AFMs, the 3D-AFMs introduced in this paper utilize a tiltable Z scanner (Fig. 1). The Z scanner can be tilted left or right to measure sidewall information accurately. Thus, our 3D-AFM can measure undercut structures regardless of the shape (Ahn et al., 2011; Cho et al., 2011).

**Fig. 2:**
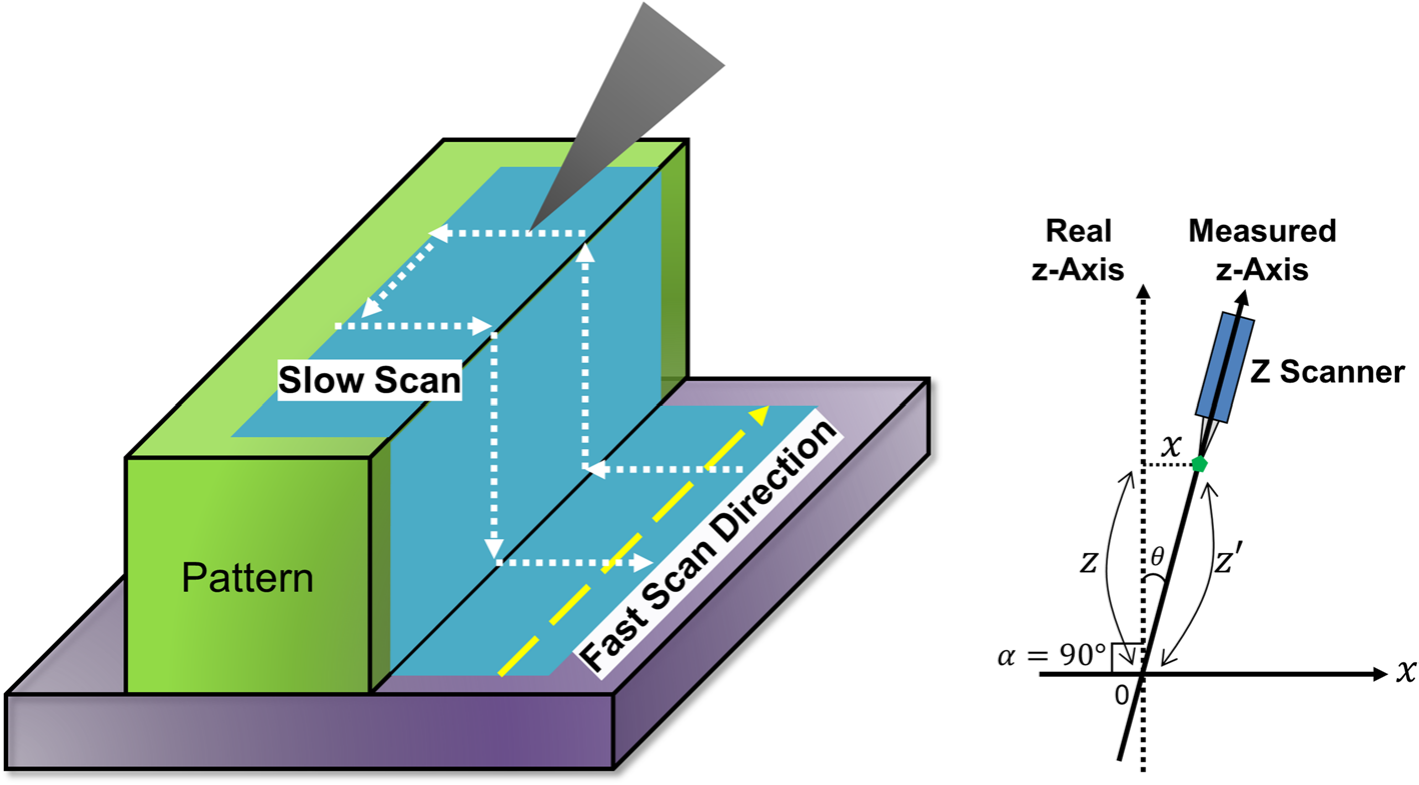
Detail of measurement position and principle of 3D-AFM Z scanner tilting sequence

As detailed in Figs. 1 and 2, the Z scanner can be tilted up to 38° left or right from the perpendicular axis (θ) and can measure the side of the pattern in the Z’ direction. Note that angle (α) is the angle of the pattern. In typical cases, the sample is vertical, and angle α will be 90°.

Since the AFM tip is attached to the bottom of the Z scanner, we can obtain the desired value X with Z’ and θ. If the Z scanner is tilted to the right by θ, we can measure the displacement of the tilted Z scanner, Z’, using the automatic AFM system. By multiplying sin θ with Z’, we get X = Z’·sin θ.

## Experimental

To analyze and confirm the repeatability and reproducibility of the 3D-AFM (Park Systems Corp., Republic of Korea), we measured the sidewall surfaces of reference samples by tilting the Z scanner 38° to the right.

**Fig. 3:**
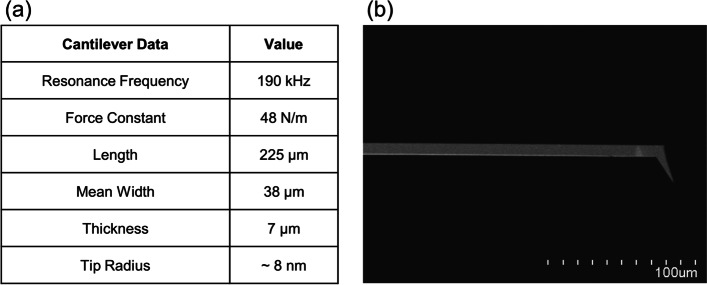
NCLR tip information. **a** Detailed specification (Park Systems Corp, 2021). **b** SEM analysis of tip side view

For the SWR measurement in Fig. 3, we used NCLR (Non-contact / Tapping™ mode - Long Cantilever - Reflex coating type, NanoWorld®, Switzerland) tip. The NCLR tip was customized to reduce hardware interference caused by tilting the Z scanner.

We measured the Metrocal wafer (MetroBoost, USA) with a continuous pattern to reduce the variation caused by non-uniform samples and measured the right side of the repeated pattern.

Since 3D-AFM is for industrial applications, we loaded the wafer sample onto the sample chuck utilizing an equipment front end module (EFEM). The tip was automatically positioned at the precise measurement location at the wafer sequentially through approaching, pattern matching, and reference scan. We then measured the sample sidewall via the set parameters.

To confirm reproducibility in non-contact mode, we repeated the roughness measurement 15 times for each of the 3 wafer dies. The time taken for image acquisition was 4 min per point. We then performed 13 wafer die measurement comparisons to analyze the SWR distribution over the entire wafer area.

**Fig. 4:**
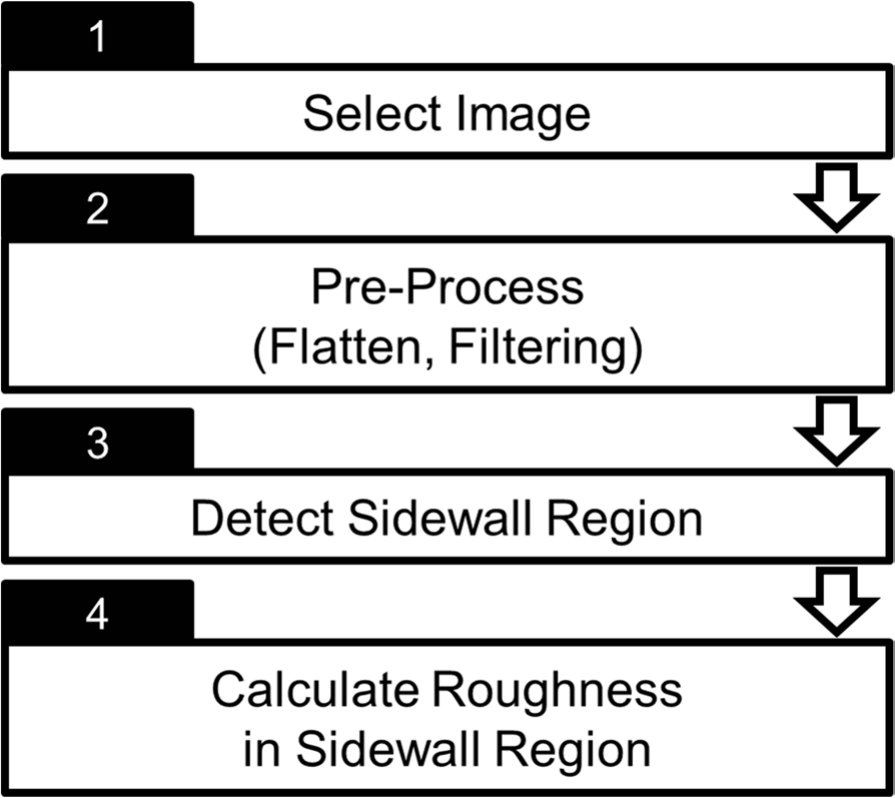
Basic algorithm of the SWR automatic analysis program

We developed an algorithm for the SWR automatic analysis program (Fig. 4), which has four processes to increase user convenience and reduce the time required for analysis. First, an image is selected for analysis. Second, the centerline is extracted from the image after Pre-Process, which consists of flattening and filtering. After a sidewall is defined in the extracted line depending on the height values, the “Detect Sidewall Region” area is cropped from the entire image. Finally, the roughness, Rq, is calculated after correcting the tilt angle of the AFM Z scanner.

## Results and discussion

The roughness of a sample is an indicator of the roughness or smoothness of the sample surface. Roughness is specified as Rq in this paper.

$$\mathrm{Rq}=\sqrt{\frac{1}{\mathrm{L}}{\int}_0^{\mathrm{L}}{\mathrm{z}}^2\left(\mathrm{x}\right)\mathrm{dx}}$$

### Rq: RMS (root mean square) roughness

**Fig. 5:**
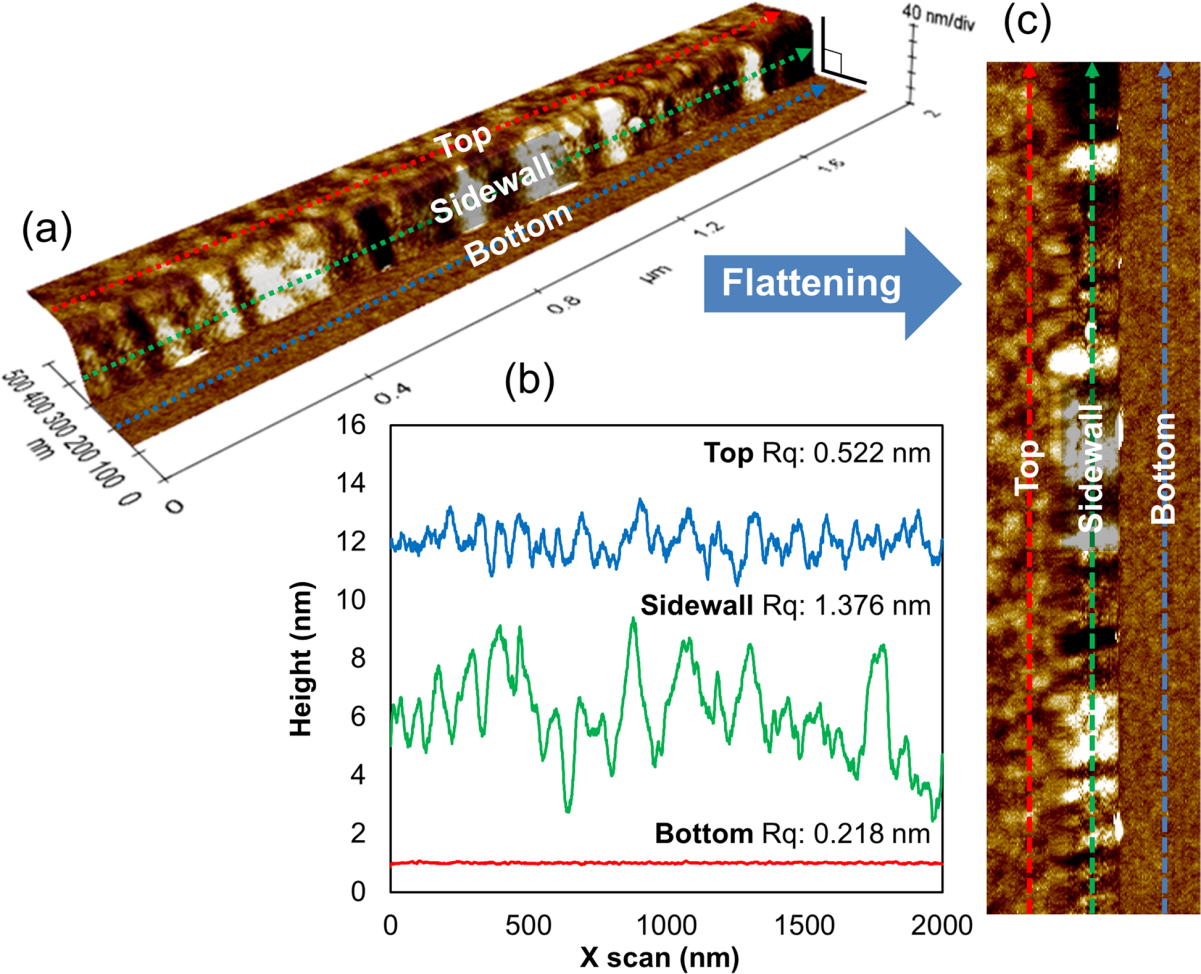
Pattern SWR measurements. **a** It is a 3D raw image overlaid flattened image. 3D raw image of a line constructed from 128 sequential line scans in the Y direction. **b** Height profiles from selected scan line of each side indicate the variation in surface roughness. **c** 2D plot of surface roughness based on first order flattening of line scan direction

The result of measuring the right sidewall of the pattern is shown as an automatically 3D rendered image in Fig. 5(a). The 3D rendered image clearly shows the difference in the roughness of each region through the topographic image, dividing the structure into top, sidewall (right sidewall), and bottom. In Fig. 5(b), line profile measurement was performed after defining a line in each region. In the profile of the measured lines, the Rq of the sidewall line was 1.376 nm, which was rougher than the top line and bottom line. In addition, the Rq of the top line is 0.522 nm, and the Rq of the bottom line is 0.218 nm, indicating a difference in roughness between the two regions.

Figure 5(c), a 2D image of Fig. 5(a), was achieved by a post-processing technique that first order flattens the measured 3D image. Then, the 2D image is divided into three regions, top, sidewall, and bottom. Since the image of the sidewall is relatively rough compared to other parts, the sidewall can also be distinguished by the difference in image contrast.

**Fig. 6:**
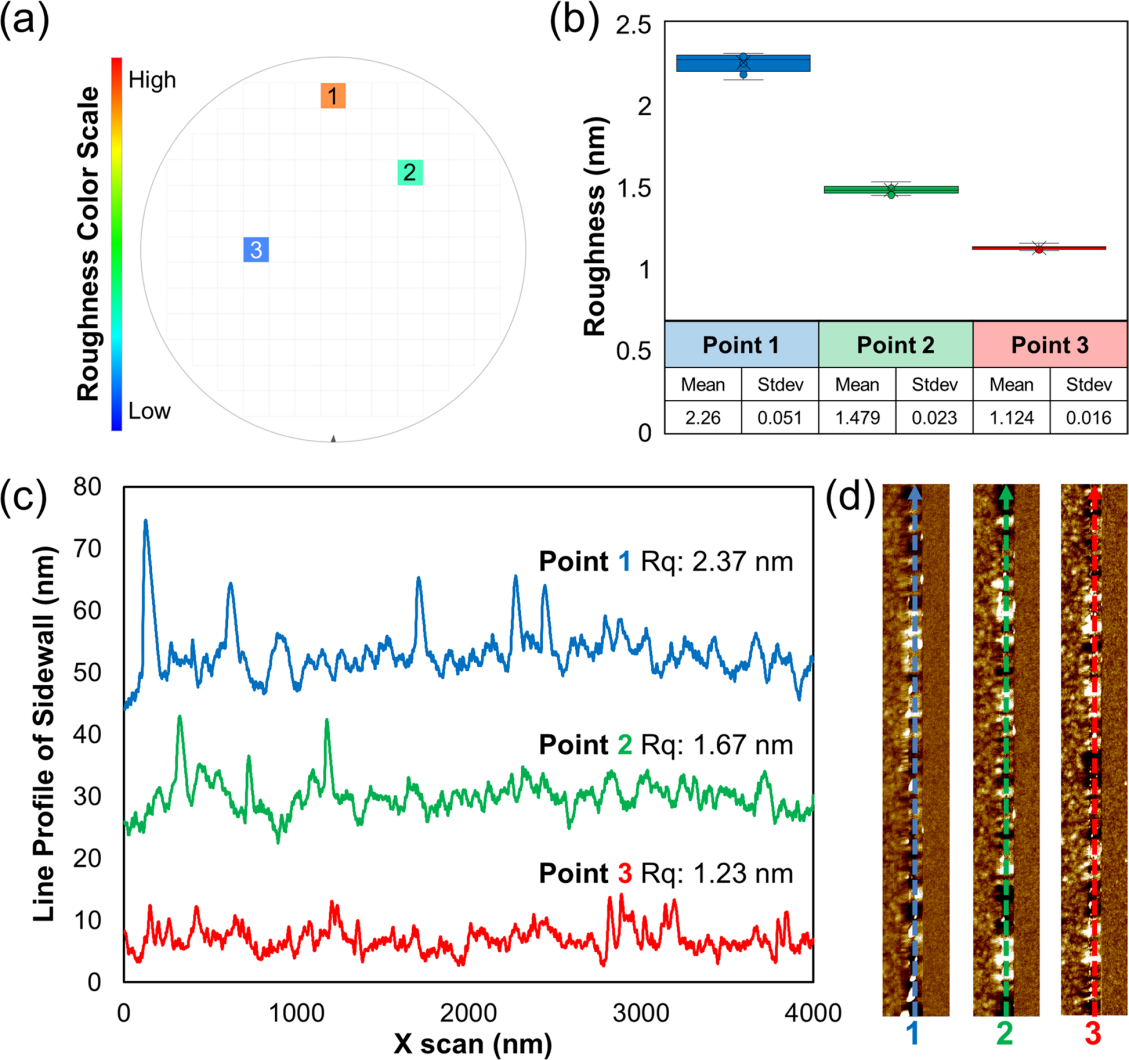
The 15 repetitive measurements of SWR on point 1, 2, and 3, randomly selected from three locations on the wafer dies. **a** The die map of the color scale regarding the average measurement result. **b** Average and standard deviation results of SWR. **c** Line profile for each measuring point. **d** AFM images after first order flattening of line scan direction

As in Fig. 6, to demonstrate the reproducibility of SWR measurements using 3D-AFM, 15 repetitive measurements were performed per identical location on point 1, point 2, and point 3, randomly selected from three locations on the wafer dies.

At point 1, the mean roughness (Rq) was measured as 2.26 nm, with standard deviation of 0.051 nm, and RMS value of 2.26%. At point 2, the mean roughness (Rq) was 1.479 nm, with standard deviation of 0.023 nm, and RMS value of 1.56%. At point 3, the mean roughness (Rq) was 1.124 nm, with standard deviation of 0.016 nm, and RMS value of 1.42%.

The measurement results found that the RMS value was close to 2% at all measurement positions in the wafer level, and the reproducibility of the measurement was proved based on these results. Accordingly, the Z scanner tilting technique for 3D measurement has high reproducibility. It has also been demonstrated that the equipment’s structural stability and measurement method are powerful (Hua et al., 2011; Hua et al., 2012).

Most importantly, even in the same pattern in a single wafer, both the topography and the roughness are different depending on the die. The SWR analysis results of measurement points 1, 2, and 3 are distributed as 2.37 nm, 1.67 nm, and 1.23 nm.

**Fig. 7:**
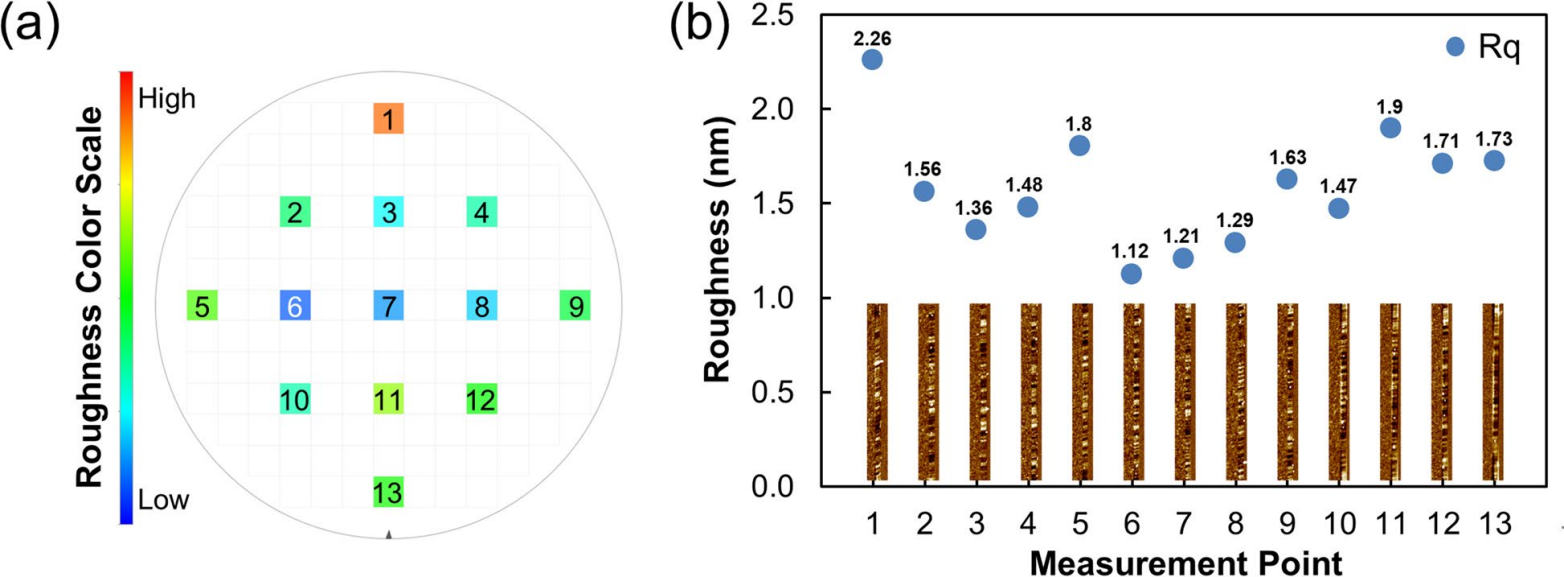
Comparison measurement of 13 points. **a** The die map of the color scale regarding to the average measurement results. **b** Rq results and AFM images after first order flattening of line scans

After confirming the reproducibility, 13 points were measured to compare the overall measurement results of the 300 mm wafer shown in Fig. 7(a). Including the center and edge of the wafer, 13 dies were selected, and the same pattern was measured once per die. As a result, the SWR, Rq, was distributed in 1.124 ~ 2.260 nm. The measured Rq for the entire wafer is distributed as shown in Fig. 7(b).

**Fig. 8:**
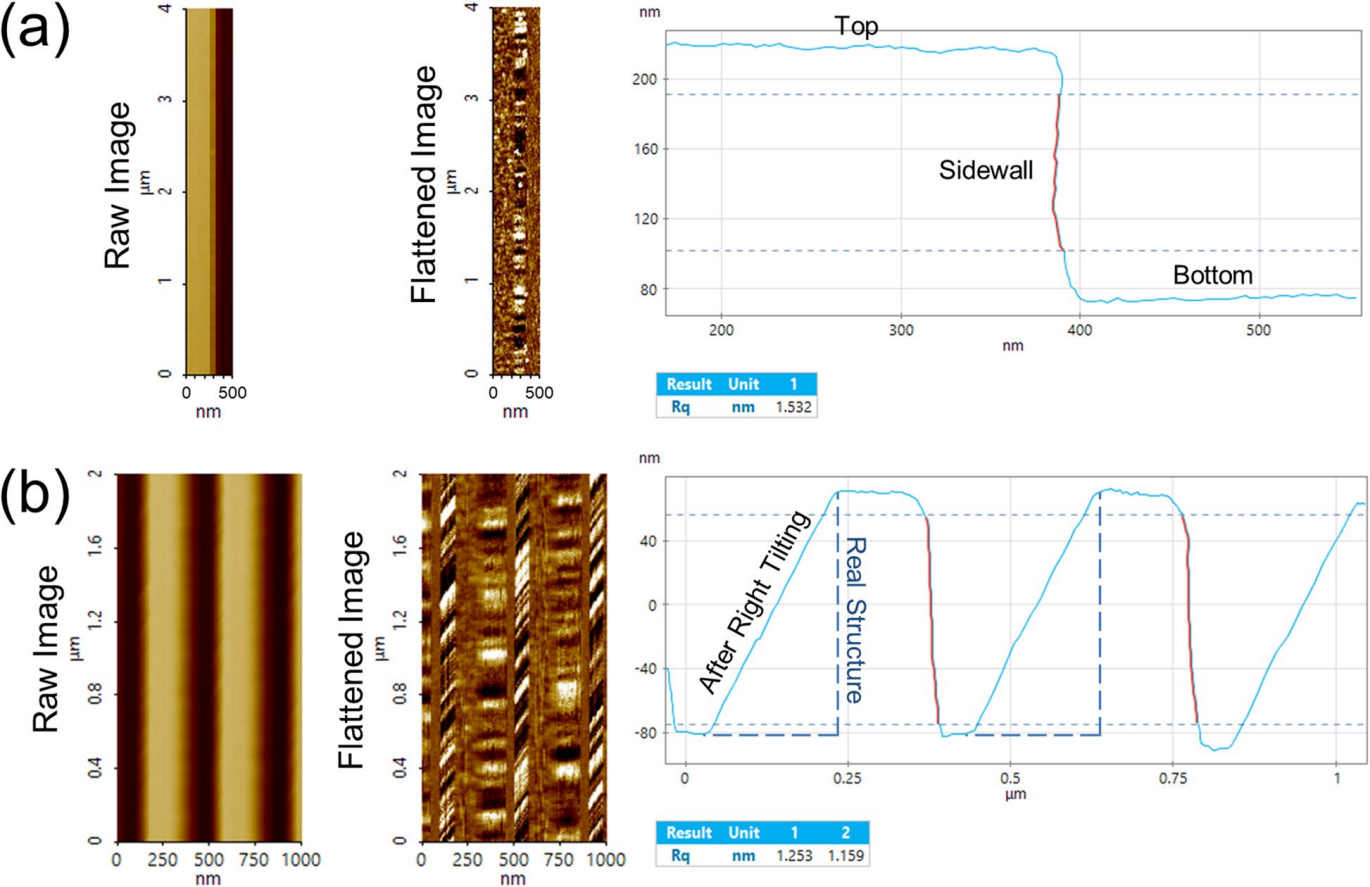
Example of SWR automatic analysis program operation screen including 1. raw image, 2. 2D plot based on first order flattening of line scans, 3. structure topography and 4. Rq results. **a** Single sidewall. **b** Multiple sidewalls

Figure 8 shows the actual analysis result of the developed SWR analysis program. When a raw image is selected, it automatically detects the sidewall with a process developed by the user, and it calculates a roughness value. In Fig. 8(a), analysis of the left sidewall is shown, and analysis of the right side is possible using the same method. Also, even in a repeated trench structure, multiple sidewalls can be analyzed, as shown in Fig. 8(b).

Furthermore, as in Fig. 8, the SWR analysis program automatically defines the maximum (top of structure) and minimum (bottom of structure) value of the structure height to define the sidewall to be analyzed at the predetermined resolution. After the sidewall is defined, the roughness of the region can be calculated.

**Fig. 9:**
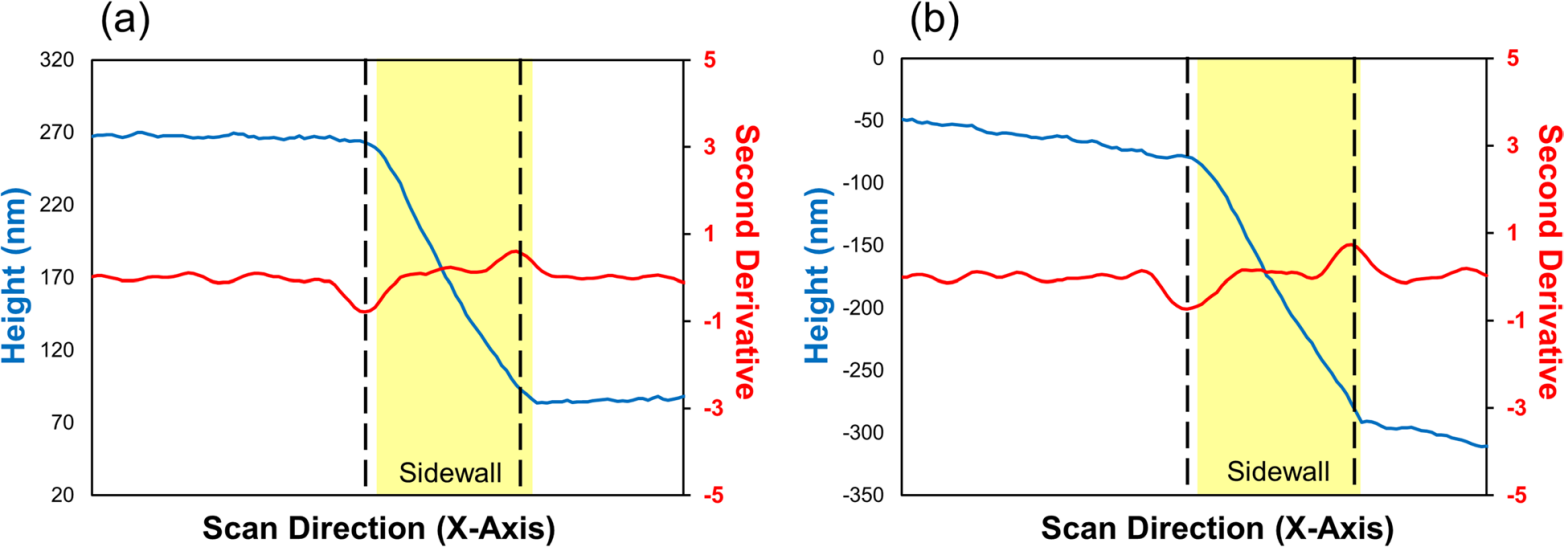
Improvement algorithm applicable to SWR automatic analysis program. **a** When the top and bottom of the structure are flat. **b** When the top and bottom of the structure are unlevel

The devised algorithm also presents a methodology for analyzing SWR by introducing the simple method of detecting an inflection point to exclude variations in the sample measurements as shown in Fig. 9. The measurement result shown in Fig. 9(a) is obtainable through the existing method of detecting sidewall by utilizing the max and min values of the sample height. In contrast, if the top and bottom of the structure are not level as in Fig. 9(b), the existing method cannot accurately detect the sidewall. Therefore, the improved algorithm enables accurate sidewall detection even in difficult-to-analyze samples or environments, as shown in both Fig. 9(a) and (b).

## Conclusions

In the semiconductor industry, the productivity of metrology tools is directly related to measurement speed and measurement reliability. Here, we implemented a fully automated 3D-AFM with an automatic SWR analysis program to measure and evaluate the SWR of vertical patterns.

Compared to previous studies, with 3D-AFM, SWR could be measured at a deeper angle, with high reproducibility of under 2%. Since this 3D-AFM is fully automated, this study showed a new possibility of industrial-level 3D-AFM usage. Unlike classic AFMs, 3D-AFM can also measure various 3D structures, including undercut samples. We expect further usage of the 3D-AFM in several challenging measurement applications at the industrial level.

The automatic SWR analysis program enabled SWR calculation without manually defining the sidewall regions. Also, with the feature of auto-flattening, the raw image could be processed automatically. The overall processing time of SWR analysis took under 2 s per image. Here, we demonstrated the possibility of implementing an automatic SWR analysis algorithm in many fabrication processes such as TSV, EUV, and GAA. Through further optimization of the algorithm and additional implementation of GPU, we expect to further reduce SWR analysis time.

## Data Availability

Not applicable.
